# First COVID-19 wave in the province of Bergamo, Italy: epidemiological and clinical characteristics, outcome and management of the first hospitalized patients

**DOI:** 10.1186/s12879-024-09034-4

**Published:** 2024-01-31

**Authors:** Bianca Maria Donida, Flavia Simonetta Pirola, Roberto Opizzi, Peter Assembergs

**Affiliations:** ASST Bergamo Ovest, Treviglio (BG), Italy

## Abstract

**Background:**

Northern Italy was the first European country affected by the spread of the SARS-CoV-2, with the epicenter in the province of Bergamo.

**Aim:**

This study aims to analyze the characteristics of patients who experienced more severe symptoms during the first wave of COVID-19 pandemic.

**Materials and methods:**

We retrospectively collected epidemiological and clinical data on patients with laboratory-confirmed wild-type SARS-CoV-2 infection who were admitted to the “ASST Bergamo Ovest” hospital between February 21 and May 31, 2020.

**Results:**

A total of seven hundred twenty-three inpatients met the eligible criteria and were included in the study cohort. Among the inpatients who survived, the average hospital length of stay was more than two weeks, with some lasting up to three months. Among the 281 non-survivors, death occurred in 50% within five days. Survivors were those whose first aid operators recorded higher oxygen saturation levels at home. The request for first aid assistance came more than one week after symptom onset, within three days in 10% of cases.

**Conclusion:**

In similar future scenarios, based on our data, if we aim to enhance the survival rate, we need to improve the territorial healthcare assistance and admit to hospitals only those patients who are at risk of severe illness requiring specialized and urgent interventions within two, three, or, at most, five days from the onset of symptoms. This implies that the crucial factor is, has been, and will be the ability of a healthcare system to react promptly in its entirety within a few days.

**Supplementary Information:**

The online version contains supplementary material available at 10.1186/s12879-024-09034-4.

## Introduction

The coronavirus disease 2019 (COVID-19) was initially reported in Wuhan City, Hubei province, China, on December 31, 2019. The cases were characterized by pneumonia ofunknown etiology [1], presenting a spectrum of clinical manifestations that typically include fever, dry cough, fatigue, and shortness of breath, often with pulmonary involvement [2]. The first case of local transmission was reported in northern Italy, specifically in the city of Codogno in the region of Lombardia [3]. Worldwide, the novel coronavirus rapidly spread, and on March 11, 2020, when more than 118,000 cases and 4,291 deaths were confirmedin 114 countries, the World Health Organization (WHO) declared the COVID-19 outbreak a pandemic [4]. In Europe, the epicenter of the pandemic was in Northern Italy, within the province of Bergamo, which is approximately 40 miles from Codogno (“Figure S1” in “Supplementary material” section). As of one year later, on December 27, 2020, authorities confirmed 137,000 deaths in Italy due to the novel coronavirus, but the estimated number of deaths could be almost twice [5]. On March 31, 2022, after over two years, the Italian government terminated the COVID-19 state of emergency and the country can gradually phase out the remaining COVID-19 measures between April 1 and December 31, 2022. The public health interventions and non-pharmaceutical measures established by the Italian government were effective in decreasing the transmission of COVID-19. Additionally, the vaccination campaign definitively contributed to achieving this millstone: as of June 2022, approximately 97% of the Italian population over 12 years and about 63% of children aged 5–11 had received at least one dose of the vaccine against SARS-CoV-2 [6]. The initial phases of the pandemic, characterized by the rapid spread of new variants of the virus, the absence of specific treatments for COVID-19, and varying levels of immunity worldwide due to different vaccine strategies, require careful consideration and make it challenging to model the future of the pandemic. In such an uncertain context, it is crucial to conduct research studies aimed at better understanding the epidemiological characteristics of patients who experienced more severe symptoms, investigating clinical differences between those who died, and evaluating the impact of health interventions on hospitalized patients. Furthermore, it is essential to utilize evidence-based knowledge to manage patients and healthcare resources effectively. This study focuses on patients with SARS-CoV-2 admitted to the “ASST Bergamo Ovest” hospital, a public health facility located in the southwest province of the epicenter area, during the first wave of the COVID-19 pandemic.

## Materials and methods

### Eligibility criteria

This study focuses on patients who were diagnosed with laboratory-confirmed wild-type SARS-CoV-2 infection and were admitted to the “ASST Bergamo Ovest” between February 21 and May 31, 2020. The “ASST Bergamo Ovest”facility comprises two hospitals: the “Treviglio-Caravaggio hospital” in the city of Treviglio, located 25 miles from Codogno and 16 miles from Bergamo, and the second one, the “Romano di Lombardia hospital”, in the city of Romano di Lombardia, situated 28 miles from Codogno and 15 miles from Bergamo. (“Figure S1” in “Supplementary material” section). Overall, the catchment area of “ASST Bergamo Ovest” covers 77 municipalities in the southwestern province of Bergamo, with a total population of 475,168 inhabitants. Throughout the outbreak, healthcare professionals at “ASST Bergamo Ovest”, during the screening and triage at the first point of contact with the healthcare system, adhered to the interim guidance published by the WHO [7], [8] to promptly recognize, isolate, and determine whether to hospitalize patients with acute respiratory illness and COVID-19 as a potential etiology. According to the case definition provided by the European Centre for Disease Prevention and Control (ECDC), the diagnosis of SARS-CoV-2 infection had to be laboratory confirmed by RT-PCR or clinically confirmed in suspected cases with radiological evidence showing lesions compatible with COVID-19 [9]. Data collection includedall ages and both sexes.

### Data collection

During the pandemic, Italian residents were discouraged from visiting hospitals as a preventive measure adopted by the Italian government to reduce the spread of the contagion and the risk of contracting the virus. Patients typically arrived at hospitals through the intervention of the public out-of-hospital emergency service, an Italian public health assistance available 24 hours a day, named SSUEM, which stands for “Servizio Sanitario di Urgenza ed Emergenza Medica”. At the hospital, clinicians conducted screening and triage of patients, following the interim guidance provided by national and international health authorities. They directed patients to the emergency room, wards, or intensive care unit based on their clinical condition and bed availability or recommended that they return home if their condition was under control. In this report, data sources include medical reports filled out in ambulances, hospital medical charts and hospital discharge records (“Scheda di Dimissione Ospedaliera”, SDO). All data were collected retrospectively, anonymized in accordance with GDPR [10] and recorded in a dedicated database. Results were obtained through aggregate analyses of the data collection.

### Investigated parameters

Patient characteristics, such as sex, age (in years), city of residence, arrival mode, time (in days) between symptom onset and hospital admission, blood type (AB0 system), oxygen saturation level while breathing ambient air (SpO_2_), deoxyhemoglobin, and the presence of pre-existing conditions (e.g., active tumour, cardiovascular disease, kidney failure, hypertension, obesity, and diabetes), are recorded. Data on non-invasive ventilation, mechanical ventilator use, or Intensive Care Unit (ICU) stay are collected, as well as information on the main medical therapies administered. These include antibiotics (with a focus on azithromycin), standard antiviral therapy, the combination of lopinavir and ritonavir tipically used in HIV management, antirheumatic disease therapies (such as chloroquine or hydroxychloroquine), corticosteroids, anticoagulants, opioids, and the IL-6 blocking antibody tocilizumab (RoActemra®), administered within the TOCIVID-19 clinical trial enrollment [11].Laboratory test results from blood samples are recorded, especially those performed at the day of hospital admission, discharge or death, and at the day of ICU admission. According to current literature [12–17], parameters concerning coagulation defects (platelet count, prothrombin time PT, partial thromboplastin time PTT, and D-dimer), immunity system disorders (levels of white blood cells WBCs), heart failure biomarkers (cardiac troponin type I hs-Tn-I and creatine kinase CK), liver failure markers (alanine aminotransferase ALT and aspartate transaminase AST) and derived markers such as AST/ALT ratio, FIB-4 and APRI index are collected. Addiotionally, data on non-specific biological molecules of tissue damage, such as lactate dehydrogenase (LDH), and markers of inflammation, such as C-reactive protein (CRP) are collected. Thromboembolic events occurring during hospitalization and codified in SDO are also recorded. The follow up (FU) time is defined as the time in days between hospital admission and hospital discharge or death.

### Ethics committee approval

This study is a retrospective epidemiological study. In Italy, observational studies without drugs must be notified to the Ethics Committee for approval. The study was submitted to and approved by the Ethics Committee of Bergamo, with reference to procedure'096/22'.

### Statistical analysis

Continuous variables are presented as mean or median with standard deviation or interquartile ranges, categorical as counts with percentages. Age and oxygen saturation as breathing ambient air SpO_2_ levels are treated both as continuous and categorical variables, with age categorized as less than 49 years, 50–69, 70–79 and 80 +,according to classes defined by Italian Health Authorities in national bulletins on COVID-19 epidemic and our population’s age distribution. SpO_2_ levels are categorized as per generally accepted standard: >95% as normal resting rate, 95 − 90% as light hypoxia and < 90% as significant hypoxia, [18–19]. Laboratory test results are treated as both continuous and binary variables (in/out of range). Receiver operating characteristics (ROC) analysis and Kernel density estimation are employed to determine the optimal cut-off values and evaluate biomarkers' probability distribution by outcome. Boxplots illustrateabsolute biomarker concentrations and changes over time, stratified per cohort and outcome. Statistical comparisons between groups use parametric and non-parametric tests as appropriate. Logistic regression is used to evaluatetherapies administration, controlling for sex, age, comorbidities, and oxygen saturation level, reporting adjusted odds ratios (aOR) and corresponding 95% confidence intervals (95% CI). Survival functions are generated using the Kaplan-Meier estimator and the Log Rank test compares differences between groups. Cox Proportional Hazard (PH) regressionevaluates covariates, with sex and age included as a priori likely confounders, and hazard ratios (HR, 95% CI) are reported. A Forest Plot illustrates the final multivariable Cox PH regression model according to Harrell’s C index. Due to the study's aim to include the entire eligible population, a priori sample size calculation was not performed. Based on estimated hospitalization and deaths’ proportion declared by Italian authorities during the study’s period [20], and considering an 0.05 type I error and two side tests, we expect to achieve 85% power or more in planned statistical analyses, even if small effect sizes based on Cohen classification would be highlight. Statistical analyses are performed using Stata Software, version 17.0 (Stata Corp LLC, Texas, USA), and the open-source R Software, version 4.1.1 (R Foundation for Statistical Computing). A two-sided *p*-value less than 0.05 is considered statistically significant. A territorial map is created using vector layers within the geographic information system open-source Q-GIS software (Version 3.16).

## Results

### Study cohort

This report focuses on patients who were admitted to the “ASST Bergamo Ovest” hospital wards between February 21 and May 31, 2020, due to COVID-19-related symptoms. The included patients had a laboratory-confirmed SARS-CoV-2 infection and were either discharged or died by the end of the follow-up period, which concluded late on the night of May 31, 2020, totaling 723 patients. The study population represents 25.8% of total hospital admissions during the study period at “ASST Bergamo Ovest”, diagnosed as suspected COVID-19 cases based on WHO interim guidance [7–8]. Please refer to the Cohort Flow Chart in “Figure S2” in “Supplementary material” section for more details.

### Patients’ characteristics, respiratory healthcare and drugs administration

**Fig. 1:**
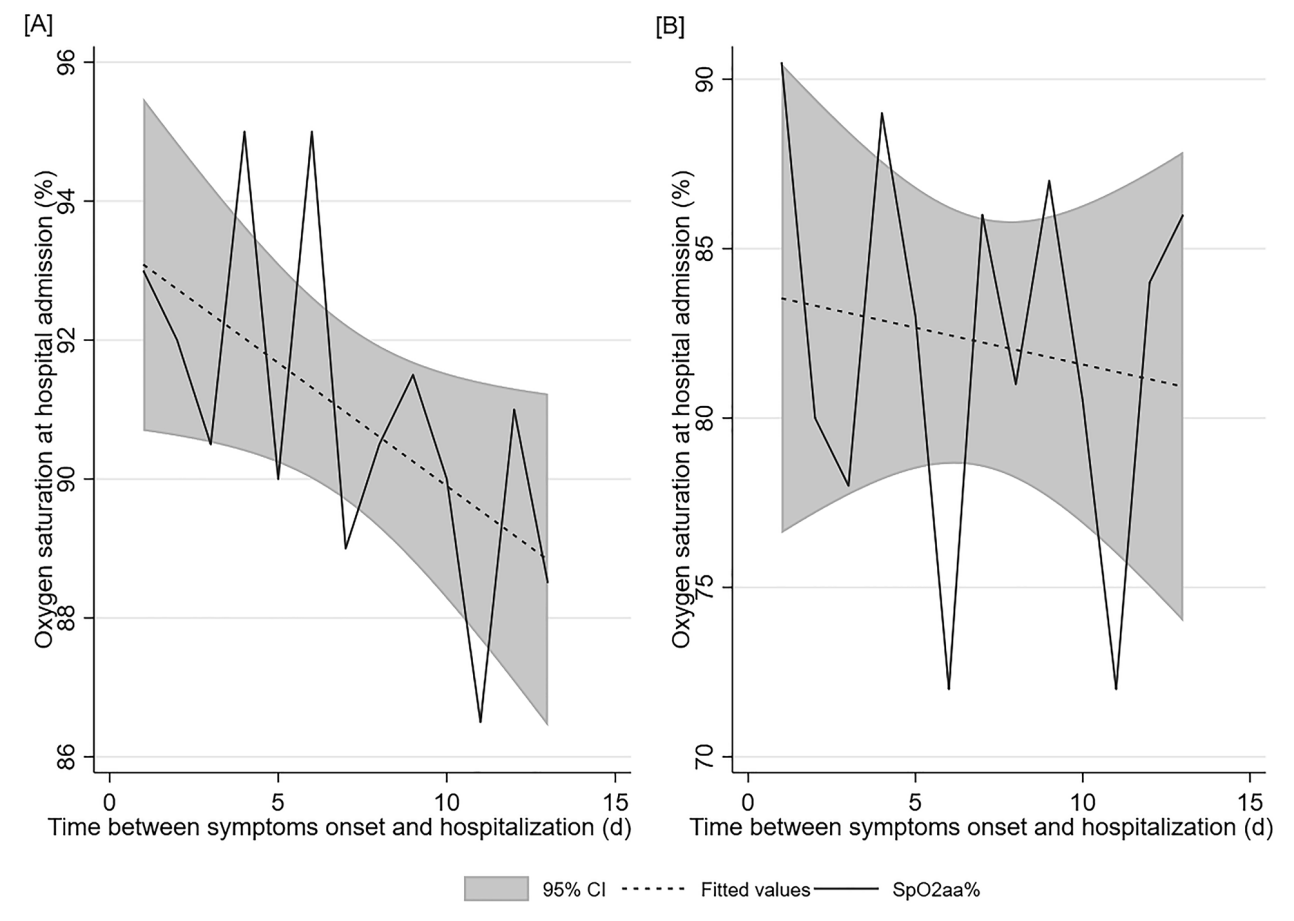
Correlation between the time (in days) waited from symptom onset to hospital admission and oxygen saturation level (% SpO_2_) while breathing ambient air at hospital admission in survivor (Panel **A**) and non-survivor patients (Panel **B**). The green line represents the average oxygen saturation levels of patients (95% CI, shown in the grey area) recorded each day (d) up to 14 days of waiting from symptom onset to hospitalization. The correlation is statistically significant in survivor patients, as detailed in the text

Of the patients, 84.8% reside in the geographical area falling within the jurisdiction of “ASST Bergamo Ovest”. Among them, 65.6% are males, resulting in a male-to-female ratio of 1.9:1. A majority of patients (90.5%) exhibit specific respiratory symptoms associated with COVID-19, and their predominant mode of hospital arrival is by ambulances (77.7%), whit 10% arriving within three days of the onset of clinical manifestation. On average, patients arrive 8.3 days (SD 5.0) after the onset of symptoms, with no significant differences by sex but statistically significant differences by age, particularly between patients under 50s and those over 80s (9.0 and 7.8 days respectively). Under a logistic regression, adjusting for patient’s sex and age, a statistically significant 5% decrease in the probability of death is observed for every day earlier arrival (*p* = 0.015^*^). The time waited between symptom onset and hospital arrival does not seem to be associate with oxygen saturation levels. Analyzing data by *exitus*, for patients hospitalized within 14 days of symptom onset (91.04%), a statistically significant correlation is found between the time waited and oxygen saturation levels at hospital arrival in patients who survived (Spearman’s rho= -0.59, *p* = 0.034^*^, “Figure 1”). The remaning 9.5% of patients contracted the infection during a planned hospital stay or arrived at hospitals for other symptoms.

**Fig. 2:**
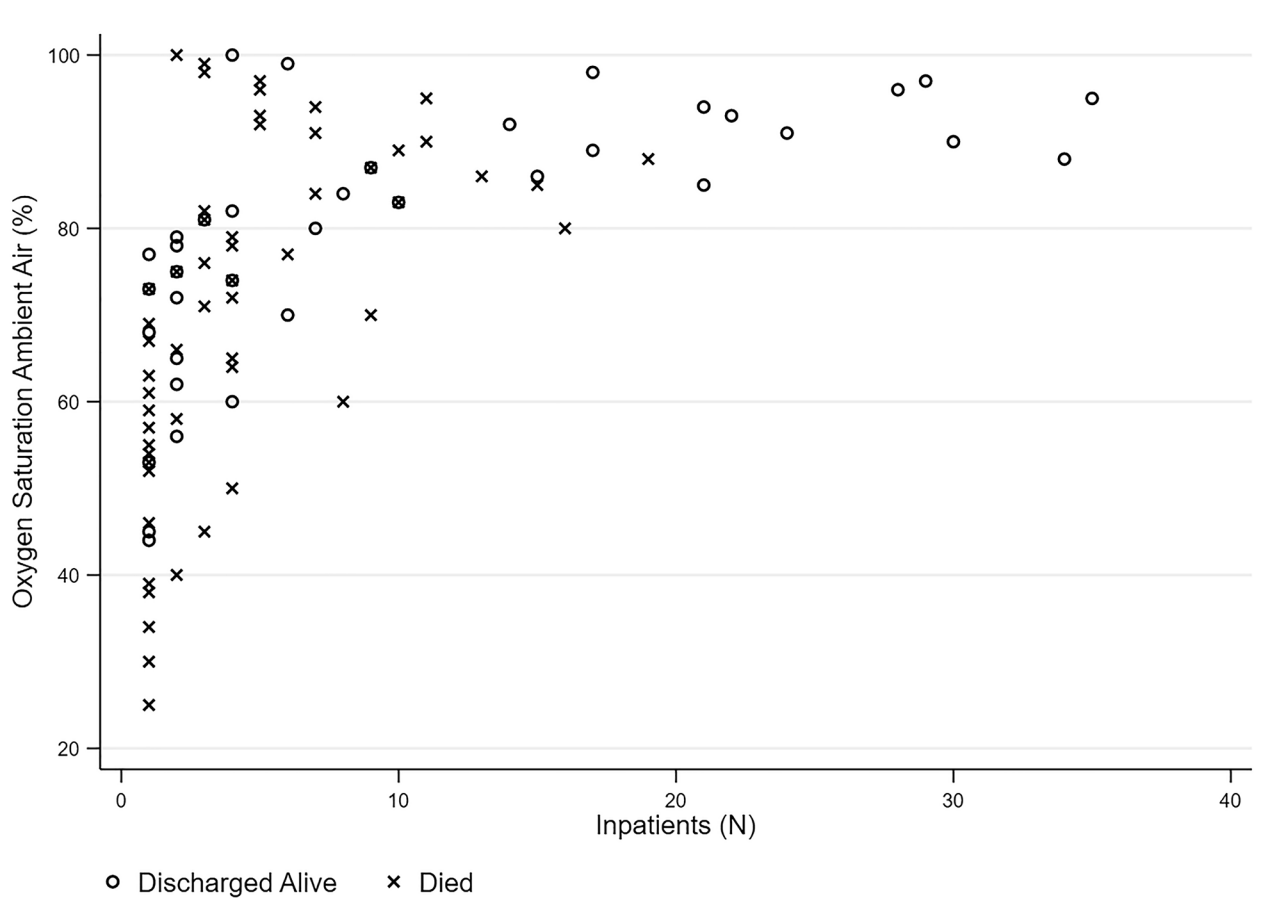
Distribution of oxygen saturation levels recorded by first aid operators before any supporting oxygen flow administration, categorized by outcome

Overall, the median age of the study population is 75 years (IQR 64–82) in femalesand 71 years (IQR 62–78) in malesrespectively (*p* < 0.001^*^). A total of 77.9% of patients have at least one concomitant disease considered in data collection: 35.0% had one comorbidity, 29.2% had two, and 13.7% had three or more, with no differences by sex. The most prevalent comorbidity is hypertension (61.8% and 63.1% in male and female, respectively, *p* = 0.744), followed by diabetes (28.9 and 21.7%, *p* = 0.037^*^), cardiovascular disease, including ischemic heart disease, coronary heart disease and myocardial infarction (21.5 and 14.9%, *p* = 0.031^*^), obesity (12.5 and 14.9%, *p* = 0.363), active tumour (11.4 and 10.4%, *p* = 0.699) and kidney failure (4.4 and 4.8%, *p* = 0.812). The distribution of the AB0 blood type system among patients is not different from the distribution among the Italian population, even adjusting for patient sex, age or *exitus*. Overall, the median oxygen saturation recorded by first aid operators before any supporting oxygen flow administration is 88% (83–94). Of the inpatients, 16% recorded a normal resting oxygen saturation above 95%, 30% recorded levels between 95 − 90%, and the remaning 54% had values lower than 90%. A scatter distribution by *exitus* is reported in “Figure 2”.

The average length of ICU stay was 24.2 days (20.0 SD) with statistically significant differences based on *exitus*. When adjusting for age, number of comorbidities, and oxygen saturation capacity, female patients appear to have a lower probability of ICU hospitalization (-56.5%, IC95% -79.1, -9.3; *p* = 0.03^*^), as well as patients of both sexes over 70 years old, showing a probability reduction of -87,6% compared to the 60–69 years old age group (IC95% -95.2,-68.0, *p* < 0.001^*^; -90.8% if over 80s, IC95% -97.1,-70.2, *p* < 0.001^*^). However, with the inclusion of laboratory tests covariatesin the model, gender loses its statistical significance.

The administration of antiviral therapy tipically used to treat HIV infection is associated with the sex of the patient (more frequent in males, *p* = 0.015^*^), age group (decreasing with increasing age, *p* < 0.05^*^) and oxygen saturation level (increasing with decreasing SpO2, *p* < 0.001^*^).

The primary class of anticoagulant employed is low-molecular-weight heparin (LMWH) administered in 68.0% of patients. Other administered anticoagulant include antiplatelets (3.8%, 24 patients), a combination of LMWH and antiplatelets (22.0%), oral anticoagulants (2.1%, 13 patients), a combination of LMWH and oral anticoagulants (3.2%, 20 patients) and direct oral anticoagulant molecules in less than 1%. Overall, anticoagulant therapies were administered in 87.4% with an association by sex (χ², *p* = 0.003^*^, aOR_male_=1.92, IC95% 1.17–3.13; *p* = 0.009^*^).

Aside of analgesic therapy administered during ICU stay, continuous opioid administration from hospital admission is statistically significant associated with sex (28.9% female and 36.3% male, *p* = 0.046^*^) and age (positive correlation, *p* < 0.001^*^). If one out of two patients over 70s received the therapy, less than one out of ten under 50s did. The association is confirmed when controlling for sex, age, oxygen saturation, and comorbidities: the probability of receiving continuous opioid therapy outside the ICU is lower in female (OR = 0.66, IC95% 0.45–0.96, *p* = 0.029^*^) and increases with age (*p* < 0.001^*^) or the number of comorbidities (OR = 1.9 IC95% 1.03–3.5, *p* = 0.040^*^).

Forty-nine patients were enrolled in the TOCIVID-19 clinical trial (EudraCT Number: 2020-001110-38) without differences by sex. Due to the enrollment inclusion criteria, we did not evaluate administration differences between groups for tocilizumab (RoActemra®).

In “Table S1” and “Table S2” reported in “Supplementary material” section, patient characteristics, respiratory healthcare, and drugs administration are summarize by *exitus* as well, using Cox PH regression models and Kaplan-Meier curves.

### Laboratory tests

**Fig. 3:**
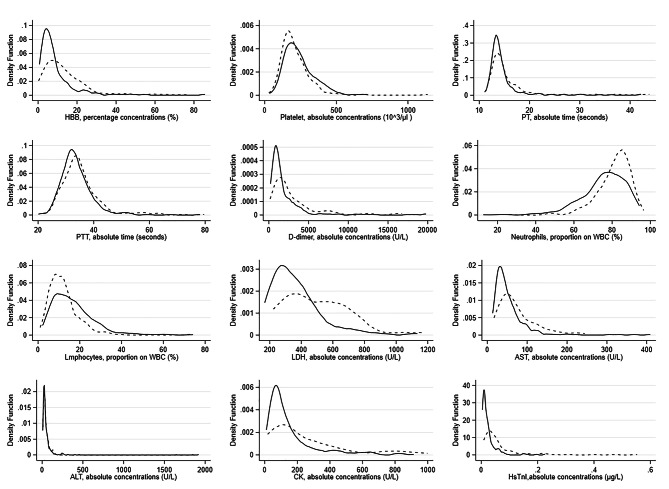
Kernel density function of laboratory tests performed on the day of hospital admission. Legend: the unit of measurements is the same as in the text, continuous line represents discharged live patients, while dotted line is for died patients. In the PT, PTT, CK, Hs Tn-I and AST graphs, outlier values (< 1%) are not included to better highlight differences in the probability distribution of biomarkers by outcome

Laboratory tests data are collected on the day of hospital admission, at the day of discharge or death, and at the day of ICU admission. Due to the low number of available data, tests collected on the day of ICU admission are not reported. It follows a description of biomarkers recorded on the day of hospital admission and their association with patients’ characteristics. Associations with the outcome are listed in “Table S1” and “Table S2” in “Supplementary material” section, Kernel density distributions by outcome are illustrated in “Figure 3”, and ROC analysis is reported in “Table S3” in the “Supplementary material” section.

#### Hypoxia

In 70% of patients, the levels of deoxyhemoglobin at hospital admission exceed the upper limit, with an overall median value equal to 7.5% (IQR 4.0-13.7) and a statistically significant association with age (*p* = 0.024^*^).

#### Coagulation defects

Median platelet count value is 205 10^3/µl (IQR 157–277, range 29-1137 10^3/µl) with an association between sexes: male patients more often have values below the lower limit, otherwise females have value above the upper limit (*p* < 0.001^*^); and with class of age (decreasing values with increasing age, *p* < 0.001^*^). The lowest PT value recorded is 11.1 s, indicating that no one has a PT value below the normal range. Overall, the PT median value is 13.9 s (IQR 13.1–15.2), with longer PT times in older patients (*p* < 0.001^*^). Median PTT value is 33.3 s (IQR 30.5–36.9), with differences by sex (*p* = 0.021^*^) and class of age (*p* = 0.002^*^). D-dimer levels were within normal range in 8.6% of patients. Overall, the median D-dimer value is 1398.5 (IQR 881-2712.5) ng/mL aside from sex, and with association by age (*p* < 0.001^*^).

#### Immunity system disorders

Data indicate changes in the proportion of WBCs, showing an increase in neutrophils and a decrease in lymphocytes, with a statistically significant association with sex. Evaluating the neutrophils and lymphocytes ratio, values greater than 9.8 [21] can double the probability of death (OR = 1.98 IC95% 1.4–2.7, *p* < 0.001^*^). Monocytes, eosinophils and basophils data are also reported in “Table 1”.

**Table 1:** Characteristics by outcome and differences between groups. Legend: star symbol is for statistically significant at *p*-value 0.05; “1” is for tests performed at the day of hospital admission, “2” is for tests performed at the day of discharge

Parameter (statistics)		Discharged Alive	Died	*p*-value
Patients (N, %)		442 (61.0)	281 (39.0)	
Gender (M/F ratio)		1.87	1.96	*p* = 0.776
Age, years (median, IQR)		68 (58–77)	77 (70–82)	*p* < 0.0001*
Symptoms to hospitalization in days (mean, SD)		8.8	7.5	*p* = 0.0004*
Oxygen saturation ambient air (median, IQR)		91 (86–95)	85 (74–90)	*p* < 0.0001*
Deoxyhemoglobin (%)		6.1 (3.5–10.0)	10.8 (6.1–17.6)	*p* < 0.0001*
Comorbidities (%)				*p* < 0.001*
	None	27.4	13.8	
	One	35.5	34.2	
	Two	28.1	31.0	
	Three or more	9.0	21.0	
Cardiovascular disease (N, %)		66 (15.0)	73 (26)	*p* < 0.001*
Diabetis (N, %)		100 (23.0)	91 (33)	*p* = 0.004*
Obesity (N, %)		53 (55.1)	43 (44.8)	*p* = 0.201
Active tumour (N, %)		42 (9.5)	38 (13.5)	*p* = 0.093
Kindey failure (N, %)		15 (3.4)	18 (6.4)	*p* = 0.059
Hypertension (N, %)		256 (58.0)	194 (69)	*p* = 0.003*
Blood type, AB0 system (%)				*p* = 0.840
	0	37.7	41.7	
	A	51.0	41.7	
	B	1.9	4.1	
	AB	9.4	12.5	
Respiratory therapy (N, %)				
	Oxygen flow	279 (63.0)	159 (57)	*p* < 0.079
	C-PAP	51 (12.0)	115 (41)	*p* < 0.001*
	VM	30 (6.8)	22 (7.8)	*p* = 0.597
ICU admission (N, %)		52 (11.8)	22 (7.8)	*p* = 0.089
ICU stay in days (mean, SD)		34.9 (3.4)	10 (2.9)	*p* < 0.0001*
Hospital stay in days (median, IQR)		14 (8–25)	6 (3–10)	*p* < 0.0001*
Drug administration (N, %)				
	Antibiotics	348 (78.7)	216 (76.9)	*p* = 0.555
	Azithromycin	302 (68.3)	150 (53.4)	*p* < 0.001*
	Standard antivirals	86 (19.5)	64 (22.8)	*p* = 0.283
	HIV antivirals	211 (47.4)	136 (48.4)	*p* = 0.862
	Antirheumatics	368 (83.3)	180 (64.1)	*p* < 0.001*
	Corticosteroids	174 (39.4)	111 (39.5)	*p* = 0.971
	Anticoagulants	388 (87.8)	244 (86.8)	*p* = 0.707
	Opioid	49 (11.1)	195 (69.4)	*p* < 0.001*
	Tocilizumab (RoActemra®)	37 (8.4)	12 (4.3)	*p* = 0.032*
PT (seconds median, IQR)^1^		13.7 (13-14.8)	14.3 (13.3–16.6)	*p* < 0.0001*
PTT (seconds median, IQR) ^1^		32.9 (30.2–36.3)	34.2 (31.2–38.1)	*p* = 0.0020*
D-dimer (ng/mL median, IQR) ^1^		1195 (760–2220)	1854 (1208–3751)	*p* < 0.0001*
Platelet count (10^3/µl median, IQR) ^1^		221 (164–292)	189 (145–255)	*p* < 0.0001*
Neutrophil proportion (% WBC median, IQR) ^1^		77.2 (69.3–84)	82.6 (76.8–86.7)	*p* < 0.0001*
Lymphocyte proportion (% WBC median,IQR) ^1^		14.9 (9.6–21)	10.8 (7.1–14.5)	*p* < 0.0001*
Monocyte proportion on WBC (% median, IQR) ^1^		6.8 (4.8–9.3)	5.7 (4.2-8.0)	*p* < 0.0004*
Eosinophil proportion on WBC (% median, IQR) ^1^		0 (0-0.4)	0 (0-0.1)	*p* < 0.0001*
Basophil proportion on WBC (% median, IQR) ^1^		0.2 (0.1–0.3)	0.2 (0.1–0.3)	*p* = 0.0055*
Neutrophils and lymphocytes ratio^1^		5.2 (3.3–8.7)	7.7 (5.4–12.0)	*p* < 0.0001*
LDH (U/L median, IQR) ^1^		330 (252.5-438.9)	468 (316–651)	*p* < 0,0001*
AST (U/L median, IQR) ^1^		41.9 (30.1–64.7)	63 (41.2–98)	*p* < 0.0001*
ALT (U/L median, IQR) ^1^		32 (21.6–54.9)	33.7 (22.6–58.9)	*p* = 0.340
APRI index (median, IQR) ^1^		0.43 (0.25–0.69)	0.70 (0.29–0.87)	*p* < 0.0001*
FIB-4 index (median, IQR) ^1^		2.2 (1.5–3.5)	2.8 (1.7–4.8)	*p* < 0.0001*
Hn Tn I (µg/L median, IQR) ^1^		0.016 (0.009–0.027)	0.043 (0.022–0.071)	*p* < 0.0001*
CK (U/L median, IQR) ^1^		103 (59–196)	216 (94–487)	*p* < 0.0001*
PT (seconds median, IQR) ^2^		13.8 (13.2–15.2)	15.9 (14.9–18.7)	*p* < 0,0001*
PTT (seconds median, IQR) ^2^		32.8 (29.5–37.4)	37.0 (32.7–43.2)	*p* < 0,0001*
D-dimer (ng/mL median, IQR) ^2^		858 (568–2115)	3,891 (678-3,564)	*p* < 0,0001*
Platelet count (10^3/µl median, IQR) ^2^		121 (195–365)	224 (154–307)	*p* < 0,0001*
Neutrophil proportion (% WBC median, IQR) ^2^		62.7 (54.6–71.5)	88.2 (83.6–92.1)	*p* < 0,0001*
Lymphocyte proportion (% WBC median, IQR) ^2^		23.8 (16.4–31.7)	6.6 (4.2–9.9)	*p* < 0,0001*
Monocyte proportion (% WBC median, IQR) ^2^		9.8 (7.9–11.5)	4.0 (2.5–6.1)	*p* < 0,0001*
Eosinophil proportion (% WBC median, IQR) ^2^		1.7 (0.7-3.0)	0.0 (0.0-0.2)	*p* < 0,0001*
Basophil proportion (% WBC median, IQR) ^2^		0.5 (0.3–0.7)	0.2 (0.1–0.3)	*p* < 0,0001*
Neutrophils and lymphocytes ratio^2^		2.6 (1.7–4.4)	13.5 (8.5–21.2)	*p* < 0,0001*
LDH (U/L median, IQR) ^2^		226 (231–335)	536 (387–745)	*p* < 0,0001*
AST (U/L median, IQR) ^2^		37 (25.3–55.0)	70.3 (30.3-101.7)	*p* < 0,0001*
ALT (U/L median, IQR) ^2^		52.4 (29.1–97.2)	39.9 (23.0-66.7)	*p* = 0.032*
APRI index (median, IQR) ^2^		0.28 (0.18–0.43)	0.63 (0.39–1.23)	*p* < 0,0001*
FIB-4 index (median, IQR) ^2^		1.18 (0.69–1.84)	3.29 (2.13–6.99)	*p* < 0,0001*
Hn Tn I (µg/L median, IQR) ^2^		0.02 (0.01–0.05)	0.03 (0.02–0.08)	*p* = 0.252
CK (U/L median, IQR) ^2^		58.2 (35.6-450.3)	94.9 (44.4-305.5)	*p* = 0.3913

#### Non-specific biomarkers of tissue damage

LDH is above the upper limit in 83.3% of patients, with a median value at hospital admission of 368.1 (277.4-513.7) U/L, without statistically significant differences between groups. Investigation on CRP at hospital admission was performed in a too few patients to draw any meaningful conclusion, data not shown.

#### Heart failure markers

CK median value is 124.7 (65.2-286.8) U/L, with higher values in male patients (*p* < 0.0001^*^). Hs-Tn I median value is equal to 0.02 (0.01–0.05) µg/L, with statistically significant differences by class of age (positive association, *p* = 0.0001^*^).

#### Liver failure markers

AST median value is equal to 47.9 U/L (IQR 32.7–73.7), with an association by sex (*p* < 0.001^*^) and age (*p* = 0.017^*^), with values over the threshold mainly reported in male patients (58.7% vs. 41.7%, *p* < 0.001^*^). ALT median value is equal to 32.8 U/L (21.9–57.4) with statistically significant differences between sexes (35.3 in male and 27.3 in female, *p* < 0.0001^*^) and by class of age (*p* = 0.0001^*^). Categorizing the variable as “in” or “out” of range, groups that more often have values over the upper limit are male (35% vs. 24.5%, *p* = 0.004^*^) and patients between 50 and 70 years old (*p* = 0.003^*^, 4 DF). The AST/ALT ratio confirms the correlation with death (OR = 2.31 IC95% 1.77–3.02, *p* < 0.001^*^). APRI index has a median value equal to 0.50 (0.29–0.87) with a statistically significant difference by sex (0.57 and 0.42 as median value in male and female respectively, *p* < 0.0001^*^). FIB-4 index collected at hospital admission has a median value equal to 2.81 (1.73–4.78), with statistically significant differences by sex (2.9 and 2.6 as median value in male and female respectively, *p* = 0.005^*^) and by class of age (positive linear correlation, *p* = 0.0001^*^). Having an elevated APRI or FIB-4 index statistically significantly increases the probability of death (OR_APRI_=2.10 IC95% 1.57–2.81, *p* < 0.001^*^ and OR_FIB−4_=1.36 IC95% 1.25–1.47, *p* < 0.001^*^).

#### Trends

When laboratory biomarkers are available at both hospital admission and discharge for the same patient, changes over time are analyzed by sex and outcome and illustrated with boxplots in “Figure S3” in Supplementary Material. According to our data, AST is the only biomarker that decreases to the normal range in a statistically significant way only in survivors. A similar but opposite trend is recorded for the platelet count that increases in a statistically significant way only in survivors, regardless of sex. Furthermore, survivors are characterized by a statistically significant increase in the proportion of lymphocytes and a reduction in neutrophils. In died patients, WBCs alteration are worse, aside from sex, with a more evident increase in neutrophils and decrease in lymphocytes. PTT values decrease in survivors and increase in died, with statistically significance differences only in male patients. A similar trend is appreciable in LDH values, even if available data are too few to lead to conclusion. Overall, the values of D-dimer remain above the range at discharge; moreover, we noticed a reduction in survivors and further increases in died patients, statistically significant in dead male and surviving female. The PT, Hs-Tn I and ALT are the biomarkers that change less between time points. CK values seem to decrease in both survivors and died patients, but data on CK are inconsistent to lead to conclusion, as previously clarified.

### Survival analysis

**Fig. 4:**
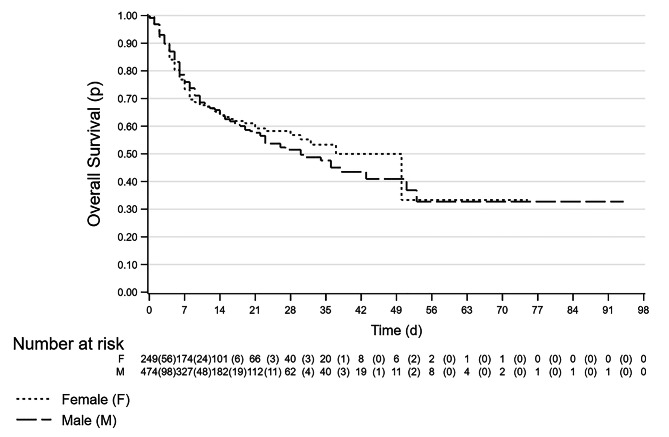
Kaplan-Meier survival estimates by sex. Legend “d” is for day and “p” is for probability, very short dash line is for female, long dash line is for male. See text for details. Note: time of follow-up is the overall LoS, and events are deaths occurred during the hospitalization

The first day of FU was set on February 21, 2020, and the May 31, 2020, marks the last day of FU. The total duration of FU spans 101 days. Individually, hospital stays ranged from one day to over three months, with the longest stay recorded at 94 days. The median length of hospital stay was ten days (mean 14.6 days, SD 12.7 days), aside from sex (10, IQR 6–20 in male and 10, IQR 6–23 in female; mean: 14.5 SD 12.8 and 14.7 SD 12.5), but depending on *exitus*: 6 days (IQR 3–10; mean: 9.0 days, SD 9.3) between who died and more than a double length of stay, LoS (14 days, IQR 8–25; mean: 18.1 days, SD 13.3) between survivors (*p* < 0.001^*^), with no differences by sexes (“Figure 4). At the end of the FU, thatwere 281deaths: 186 male and 95 female patients (p = 0.776), disclosing a lethality rate of 38.9% among frail patients during the first pandemic wave. The 75.8% of deaths occurred within ten days of hospital admission (73.7% in male and 80% in female, p = 0.665), with 18.5% occurring within two days (17.7% in male and 20.0% in female, p = 0.703). Overall, the median survival time was about 34 days (26–50): 30 days (23–43) in male and 37 days (28-nr) in female, withno statistically significant differences between sexes. Kaplan Meyer survival analyses are summarize in “Table S2” in “Supplementary material” section. Cox PH regression analysis on survival is reported in “Table S1” in “Supplementary material” section as a single model on covariate, adjusted for sex and age. Multivariable Cox PH model, characterized by Harrell’s C index of 0.92, is represent with a Forest Plot in “Figure S4” in the “Supplementary material” section. To avoid redundancy of information we only considered the FIB-4 index to disclose liver failure, categorizing the values of the marker according to the literature [22]. Due to the low rate of laboratory tests, we could not include LDH in the analysis, despite its association with the risk of death, as demonstrate in the univariate analysis.

### Thromboembolic events

Overall, thromboembolic events were reported in 124 SDOs (17.2%), with no association by sex and *exitus*, but with a statistically significant association with the age of the patient, indicating a lower risk of events in younger patients (*p* = 0.008^*^). Under sex and age equality, the risk of thromboembolic events during hospital stays increased by 74% in obese subjects (*p* = 0.043^*^) and decreased by 37% in patients who received oxygen supplementation (*p* = 0.025^*^). Otherwise, still under sex and age equality, preventive therapy for deep venous thrombosis did not appear to decrease the likelihood of thromboembolic events, even after controlling for other administered drugs. Of the specified events (*N* = 107), the majority affected the heart (75.4%), followed by the lung (12.3%), the central nervous system (4.9%) and an arm or leg (3.3%). There was a statistically significant association between the site of onset and the *exitus* (*p* = 0.010^*^). Specifically, events predominantly affected the heart tissue in patients who died (92% versus 65%), while the lung or central nervous system were more common in survivors (19% vs. 2% and 6.8% vs. 2.1% respectively).

## Discussion

This study focuses on patients infected with SARS-CoV-2 who were hospitalized at “ASST Bergamo Ovest”, a public health facility located in the southwest geographical area, which was the European epicenter of the COVID-19 pandemic during the first wave in Italy. Although standardized incidence rates are needed to clearly define the phenomena, it is evident that male patients were more numerous and older than female ones. Overall, hospitalized patients were elderly and frail, with a median age above 70 years, and they often had at least one comorbidity. Our results suggest that the virus divided our cohort into two groups based on oxygen saturation capacity straight from the infection, although the mechanism remains unknown. The average time waited toseek health assistance was a week longer from symptoms’ onset, and it correlated with oxygen saturation level, pulmonary capacity, and survival, aside from sex. Upon hospital admission, almost 70% of patients had a SpO_2_ value below 95%. Seventy-four patients were admitted to the ICU, and the ICU stay was three times longer for those who survived. A strong statistical association was found between the probability of ICU admittion and patient’s age. According to our data, controlling for sex, age, oxygen saturation and comorbidities, the administration of azithromycin dihydrate (among antibiotics), antirheumatic disease therapy, and anticoagulants statistically reduced the risk of death. The probability to die also decreased statistically for patients enrolled in the TOCIVID-19 clinical trial (EudraCT Number: 2020-001110-38) [11], who received the biological therapy tocilizumab (RoActemra®), an immunosuppressive drug belonging to the class of drugs known as Interleukin-6 blockers, commonly used to treat adults with moderate to severe rheumatoid arthritis.

Laboratory data on patients with wild type SARS-CoV-2 infection revealed differences by sex, with male patients showing worse alterations form normal ranges compared to females, both at hospital admission and discharge. Overall, detected laboratory alternations included a reverse proportion in WBCs, higher levels of LDH, D-dimer, CK, AST and Hs-Tn-I levels and shorter PT and PTT times.

Overall, hospital length of stay ranged from few days to more than three months. Median hospital LoS was ten days, aside from sex, but depending on *exitus*, with a minimum of double LoS between who survived. The lethality rate is approximately 40%, regardless of sex. More than two-third of deaths occurred within ten days of hospital admission, with about a quarter occuring within two days, aside from sex. According to the multivariable PH Cox regression model, the included covariates that statistically seemed to increase the risk of death were higher levels of liver biomarkers and continuous opioid administration. Otherwise, corticosteroids administration, oxygen flow support, and ICU stay appeared to decrease the risk of death under other covariates' equality. 

Notably, the incidence of thromboembolic events, lower among younger inpatients, seems to be directly associated with obesity, mainly involving heart tissue in those who died.

Our data have limitations as they were retrospectively collected, focusing only on patients who reached the Emergency Department (ED) at “ASST Bergamo Ovest” during the first wave of the COVID-19 pandemic. Our analyses are focused on symptoms and hospitalizations of the initial COVID-19 cases in the area.

## Conclusion

Authors, in preparing this work, primarly aim to convey a lesson learned through experience, serving as an evaluative starting point for emergency management during an outbreak of an emerging infectious disease. Such situations are characterized by overcrowding in ED, affecting resources availability and the quality of care. This, in turn, results in increased mortality and morbidity, particularly in the absence of specific therapies, as seen during the COVID-19 outbreak. Learning how to ensure critical services in a timely manner is crucial. Evaluating evidence-based prognostic factors is essential for planning intervention strategies to improve clinical management and patient outcome, and for allocating scarce health resources properly.

When evaluating the results, it should be considered that the health system was under the immense pressure due to the pandemic. Despite Italy's sudden efforts to increase overall hospital and ICU bed capacity, including setting up camp hospitals, converting fairground spaces, and cancelling elective surgeries, the high infection rate and resulting hospitalization rate still lead to an imbalance between actual clinical need and healthcare resources. Furthermore, the long-required stay for COVID-19’ patients posed an additional challenge during the outbreak of the pandemic. In this emergency scenario, where criteria for ICU admission and discharge may be necessary, the “Italian Society of Anesthesia Analgesia Resuscitation and Intensive Care” (SIAARTI) published operational recommendations and ethical consideration on March 6, 2020, to support the clinicians involved in the care of critically ill patients [23]. These recommendations advised clinicians not necessarily to follow the traditionally “first come, first served”ICU admission policy. Instead, they were urged to carefully evaluate age, comorbidities, and functional status when allocating limited resources. The document also suggested that clinicians consider the ceiling of care if a patient is deemed “not appropriate for intensive care” and decisions like “do not intubate” should be documented in medical charts in advance. Additionally, according to SIAARTI guidelines, palliative sedation should be evaluated as necessaryin patient with hypoxia and disease progression. To care for patients, clinicians followed interim guidance from the WHO [24–25] and local health authorities, such as AIFA guidelines [26], and providing healthcare services appropriately.

This study clearly highlights the need to improve territorial healthcare assistance and to admit to hospitals only frail patients at risk for severe illness requiring specialized and urgent interventions, within two or three, maximum five days from symptoms onset. This underscores that crucial factor is, has been, and will be the ability of a healthcare system to respond promptly in its entirety within a short time frame.

### Electronic supplementary material

Below is the link to the electronic supplementary material.

Supplementary Material 1

## Data Availability

The dataset analyzed during the current study is available from the corresponding author on reasonable request.

## Abbreviations

AIFA: Agenzia Italiana del Farmaco
ALT: Alanine Aminotransferase
APRI: Platelet Ratio Index
ASST: Azienda Socio-Sanitaria Territoriale
AST: Aspartate Transaminase
C-PAP: Continuous Positive Airway Pressure
CI: Confidence Interval
CK: Creatine Kinase
COVID-19: Coronavirus Disease 2019
CRP: C-Reactive Protein
DF: Degree of freedom
ECDC: European Centre of Disease Prevention and Control
ED: Emergency Department
FIB-4: Fibrosis-4 Index
FU: Follow-up
GDPR: General Data Protection Regulation
HR: Hazard Ratio
hs-Tn I: Cardiac Troponin type I
ICU: Intensive Care Unit
LDH: Lactate Dehydrogenase
LoS: Length of stay
N: Number
Nr: Not reached
OR: Odds Ratio
PT: Prothrombin Time
PTT: Partial Thromboplastin Time
RT-PCR: Reverse Transcriptase Polymerase Chain Reaction
SARS-CoV-2: Severe Acute Respiratory Syndrome Coronavirus 2
SDO: Scheda di Dimissione Ospedaliera
SpO_2_: Oxygen Saturation
SSUEM: Servizio Sanitario di Urgenza ed Emergenza Medica
U/L: units per litre
WBCs: White Blood Cells
WHO: World Health Organization
µl: microliter (International System Unit)
