# Supplementary material for: First COVID-19 wave in the province of Bergamo, Italy: epidemiological and clinical characteristics, outcome and management of the first hospitalized patients
Source: BMC Infect Dis. 2024 Jan 31;24:151. doi: 10.1186/s12879-024-09034-4 (PMC10829314; doi:10.1186/s12879-024-09034-4)
Supplement: Supplementary file 1 — Supplementary Material 1 [file 12879_2024_9034_MOESM1_ESM.pdf]

Supplementary Material – Tables

Covariate	Data Availability (%)	Covariate Level	aHR	95% CI		p-value
Sex	100	Female	0.82	0.64	1.05	p=0.119
Class of age (years)	100	<50	0.00	0.00		p=1.000
		50-59	0.20	0.10	0.38	p<0.001*
		60-69	0.71	0.52	0.98	p=0.038*
		80+	1.18	0.90	1.55	p=0.234
Comorbidities (N)	100	One	1.02	0.69	1.49	p=0.936
		Two	1.13	0.77	1.66	p=0.547
		Three+	1.63	1.07	2.47	p=0.022*
Hypertension			1.04	0.81	1.35	p=0.747
Diabetis			1.25	0.98	1.61	p=0.078
Obesity			1.42	1.02	1.98	p=0.036*
Cardiovascular disease			1.06	0.81	1.39	p=0.681
Kidney failure			1.38	0.86	2.23	p=0.185
Active tumour			1.16	0.82	1.63	p=0.403
Oxigen Saturation level	90	95-90%	1.31	0.76	2.25	p=0.339
		<90%	3.25	2.00	5.27	p<0.001*
Respiratory support	100	Oxygen	1.69	1.08	2.66	p=0.023*
		C-PAP	5.45	3.44	8.64	p<0.001*
		MV	2.71	1.47	5.02	p=0.001*
ICU stay	100		0.73	0.46	1.16	p=0.181
Antibiotics	100		0.78	0.59	1.03	p=0.079
Azithromycin	100		0.58	0.46	0.73	p<0.001*
Standard antivirals	100		1.22	0.92	1.62	p=0.160
HIV antivirals	100		1.03	0.81	1.31	p=0.815
Antirheumatics	100		0.38	0.29	0.48	p=0.001*
Corticosteroids	100		0.68	0.53	0.87	p=0.002*
Anticoagulants	100		0.55	0.39	0.78	p=0.001*
Opioids	100		4.58	3.54	5.91	p=0.001*
Tocilizumab (RoActemra®)	100		0.51	0.28	0.91	p=0.022*
Deoxyhemoglobin, %	58	<1.4	0.988	0.381	2.567	p=0.981
		>4.9	2.152	1.437	3.223	p=<0.001*
Platelet count, 10^3/µl	96	<150	1.282	0.978	1.681	p=0.072
		>400	0.442	0.226	0.866	p=0.017*
Prothrombin time, seconds	91	>170	1.240	0.964	1.595	p=0.094
Partial thromboplastin time, seconds	89	<24	0.915	0.226	3.702	p=0.901
		>38	1.236	0.925	1.650	p=0.152
D-dimer, U/L	63	>500	2.439	0.900	6.612	p=0.08
Neutrophils/ lymphocytes ratio	95	>9.8	1.407	1.094	1.809	p=0.008*
Lactate dehydrogenase, U/L	23	>250	2.686	0.975	7.394	p=0.056
Aspartate transaminase, U/L	77	>47	2.145	1.585	2.904	p=<0.001*
Alanine aminotransferase, U/L	96	>47	1.288	0.991	1.674	p=0.059
Creatine kinase, U/L	65	<27	0.837	0.263	2.659	p=0.762
		>170	2.278	1.675	3.099	p=<0.001*
Cardiac troponin type I, µg/L	46	>0.014	3.300	1.771	6.148	p=<0.001*

Table S1. *Single Cox PH regression models controlling for sex and age. Example: 1st line: result for gender is controlled for age, 3rd line: results for comorbidities are adjusted for sex and age. Legend: aHR is for adjusted Hazard Ratio (95% CI). The reference covariates' level are: male, 70-79 class of age, absence of comorbidities, >95% oxygen saturation level recorded by first operators before any supporting oxygen flow administration, no therapy administration and biomarker's in-range value for laboratory tests performed at the day of hospital admission, in details: deoxyhemoglobin: 1.4-4.9%, platelet count:150-400 10^3/µl, PT:9.5-13.8 seconds, PTT: 24-38 seconds, D-dimer:<500 U/L, neutrophils lymphocytes ratio:<9.8 [21], lactate deydrogenase:135-250 U/L, AST and ALT:<47 U/L, creatine kinase:27-170 U/L, cardiac troponin Hs-Tn-I: <0.014 µg/L; "pts" is for patients and star's symbol is for statistically significant at p<0.05.*

Parameter	Group	Patients (N)	Events (N)	Events (%)	mOS (days)	95% CI		Log Rank Test
Gender	Male	474	186	39.2	30	23	43	p=0.784
	Female	249	95	38.2	37	28	nd	
Class of age (years)	<50	38	0	0.0	nr	nd	nd	p<0.0001*
	50-59	100	10	10.0	nr	nd	nd	
	60-69	163	56	34.4	nr	23	nd	
	70-79	235	114	48.5	23	16	34	
	80+	187	101	54.0	17	10	32	
Comorbidities (N)	None	160	39	24.4	50	27	nd	p<0.0001*
	One	253	96	37.9	36	23	nd	
	Two	211	87	41.2	32	20	nd	
	Three+	99	59	59.6	11	8	23	
Hypertension	No	273	87	31.9	37	30	nd	p=0.025*
	Yes	450	194	43.1	30	19	51	
Diabetis	No	532	190	35.7	37	30	nd	p=0.008*
	Yes	191	91	47.6	23	14	38	
Obesity	No	627	238	38.0	36	26	50	p=0.080
	Yes	96	43	44.8	30	14	53	
Cardiovascular disease	No	584	208	35.6	50	31	nd	p=0.009*
	Yes	139	73	52.5	21	13	30	
Kidney failure	No	690	263	38.1	36	27	50	p=0.181*
	Yes	33	18	54.5	20	7	nd	
Active tumour	No	643	243	37.8	36	27	50	p=0.142*
	Yes	80	38	47.5	23	8	nd	
Oxigen Saturation level	>95%	102	18	17.7	51	28	nd	p<0.0001*
	95-90%	192	46	24.0	36	31	nd	
	<90%	352	190	54.0	14	10	23	
Respiratory support	None	139	22	15.8	nr	32	nd	p<0.0001*
	Oxygen	392	130	33.2	50	36	nd	
	C-PAP	140	107	76.4	6	6	8	
	MV	52	22	42.3	51	17	nd	
ICU stay	No	649	259	39.9	31	23	43	p=0.006*
	Yes	74	22	29.7	52	30	nd	
Antibiotics	No	159	65	40.9	28	17	nd	p=0.170
	Yes	564	216	38.3	36	26	50	
Azithromycin	No	271	131	48.3	17	12	28	p<0.0001*
	Yes	452	150	33.2	43	34	nd	
Standard antivirals	No	573	217	37.9	36	26	50	p=0.363
	Yes	150	64	42.7	30	16	nd	
HIV antivirals	No	376	145	38.6	37	26	51	p=0.056#
	Yes	347	136	39.2	30	23	nd	
Antirheumatics	No	175	101	57.7	8	6	11	p<0.0001*
	Yes	548	180	32.8	43	34	nd	
Corticosteroids	No	438	170	38.8	30	21	50	p=0.003*
	Yes	285	111	38.9	43	30	nd	
Anticoagulants	No	91	37	40.7	23	9	nd	p=0.006*
	Yes	632	244	38.6	36	28	50	
Opioids	No	479	86	18.0	nr	51	nd	p<0.0001*
	Yes	244	195	79.9	9	7	10	
Tocilizumab (RoActemra®)	No	674	269	39.9	30	23	50	p=0,003*
	Yes	49	12	24.5	43	38	nd	

Table S2. *Survival analysis. Patients (as N) and events (as N and %) within groups are reported. Median survival time in days estimates by Kaplan-Meier curves (mOS, IC95%) and differences between groups obtained by Log Rank test are reported as well. Legend: star is for "statistically significant at p-value<0.05". Significant associations are confirmed controlling for sex, age, oxygen saturation level and comorbidities as well.*

Laboratory test	AUC	95% CI	Threshold	Specificity (%)	Sensitivity (%)
Cardiac troponin type I, µg/L	0.7867	0.7383-0.8351	0.03	75.7%	70.2%
FIB-4 index	0.7381	0.6952-0.7810	3.63	76.8%	60.0%
Lactate dehydrogenase, U/L	0.7102	0.6306-0.7898	458.65	83.3%	51.5%
APRI index	0.6876	0.6416-0.7335	0.48	56.9%	71.5%
AST/ ALT ratio	0.6813	0.6335-0.7291	1.80	83.9%	47.0%
Deoxyhemoglobin, %	0.6729	0.6203-0.7254	7.25	61.5%	70.1%
Aspartate transaminase, U/L	0.6726	0.6261-0.7191	57.25	70.3%	57.5%
D-dimer, U/L	0.6656	0.6093-0.7218	1384.0	58.7%	70.4%
Creatine kinase, U/L	0.6609	0.6087-0.7131	167.25	72.1%	56.3%
Neutrophil proportion on WBC, %	0.6508	0.6097-0.6920	79.15	58.4%	68.4%
Neutrophils/ lymphocytes ratio	0.6503	0.6092-0.6914	5.38	52.5%	75.1%
Lymphocyte proportion on WBC, %	0.6441	0.6028-0.6854	14.45	52.3%	74.7%
Prothrombin time, seconds	0.5974	0.5524-0.6424	14.45	69.8%	46.9%
Platelet count, 10^3/µl	0.5932	0.5502-0.6363	206.0	55.7%	59.9%
Monocyte proportion on WBC, %	0.5796	0.5359-0.6233	6.25	57.7%	57.6%
Partial thromboplastin time, seconds	0.5717	0.5262-0.6171	32.35	44.9%	68.2%
Alanine aminotransferase, U/L	0.5217	0.4772-0.5662	23.70	32.2%	74.4%
Basophil proportion on WBC, %	0.4390	0.3963-0.4816	1.35	99.3%	0.7%
Eosinophil proportion on WBC, %	0.3952	0.3581-0.4323	nd		

Table S3. *Receiver operating characteristics by outcome. Results for laboratory tests performed at the day of hospital admission are listed in decreasing order according to the value of the related Area Under the Curve (AUC, 95% CI). Suggested biomarker's threshold, specificity and sensitivity percentages are reported as well.*

Supplementary Material – Figures

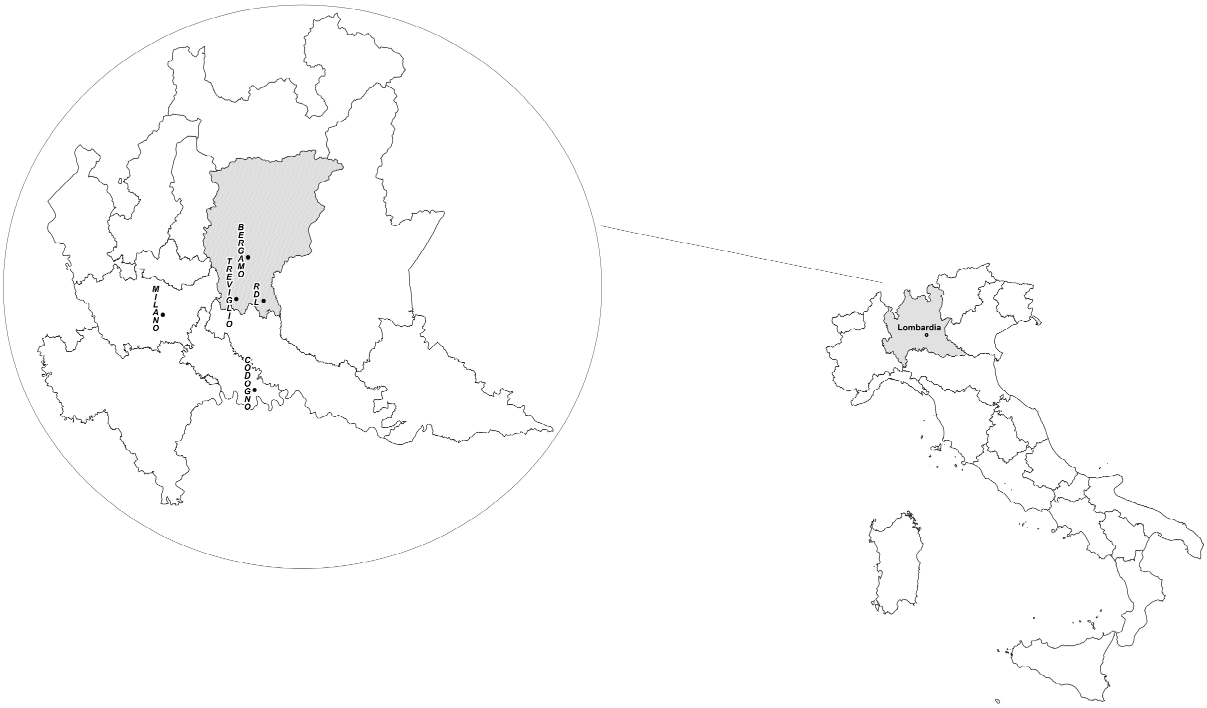

Figure S1. *Graphical maps obtained using vector layers in the geographic information system Q-GIS software (Version 3.16). The region of Lombardia, the light grey area in the map of Italy on the right and zoomed in the circle on the left, is one of the twenty administrative regions of Italy. It expands all the way up for 9,206 square miles in the northern-central part of the country. It has a population of about 10 million people, corresponding more than one-sixth of all country population, and its capital is the city of Milano. Codogno is a small city in the province of Lodi in south of the Lombardia's region, and it is far about 40 miles from the city of Bergamo. Bergamo's city is the mayor city of the province of Bergamo, the European epicenter of the pandemic: the area filled in grey in zoomed map of Lombardia on the left. The two cities where the two hospitals that constitute the "ASST Bergamo Ovest" health facility are located are Treviglio and Romano di Lombardia. These cities are both belonging to the province of Bergamo, and they are respectively 16 and 15 miles from the city of Bergamo and 25 and 28 miles from Codogno. Legend: "RDL" is for Romano di Lombardia.*

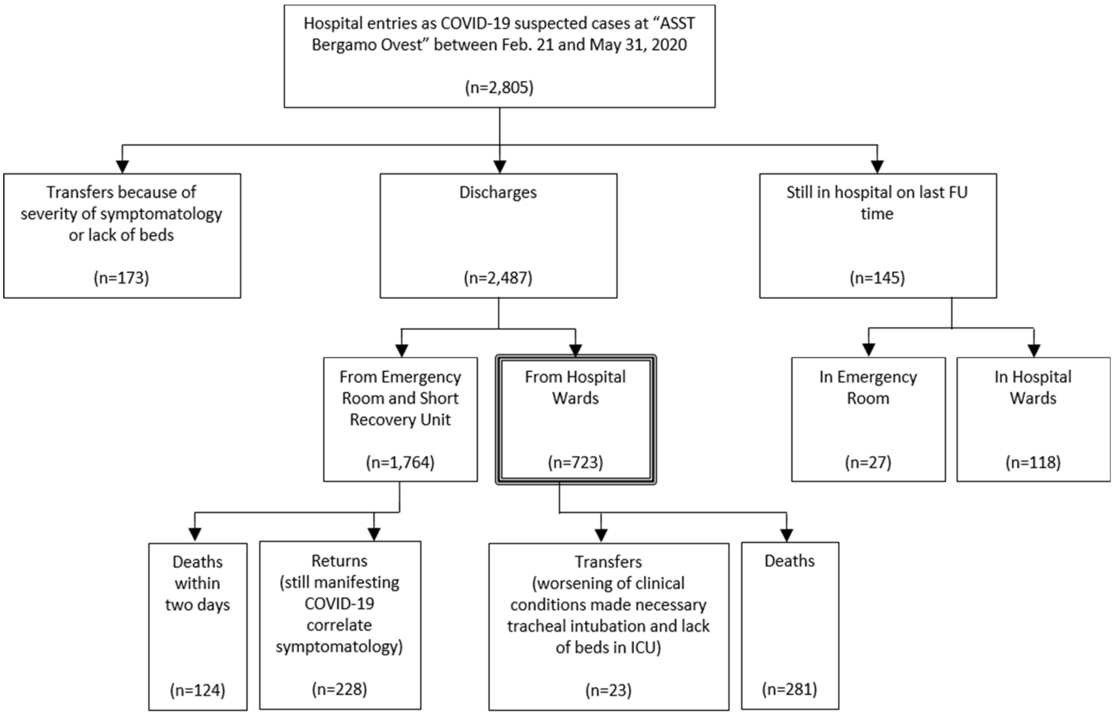

Figure S2. *Cohort flow chart. This report focus on patients who were admitted to the "ASST Bergamo Ovest" hospital wards between February 21 and May 31, 2020, because of symptomatology COVID-19 correlate, who had a laboratory confirmed SARS-CoV-2 infection and were discharged within the end of the follow up, on late night of May 31, 2020, for a total of 723 patients. In the chart, the Study's cohort corresponds to the double line box.*

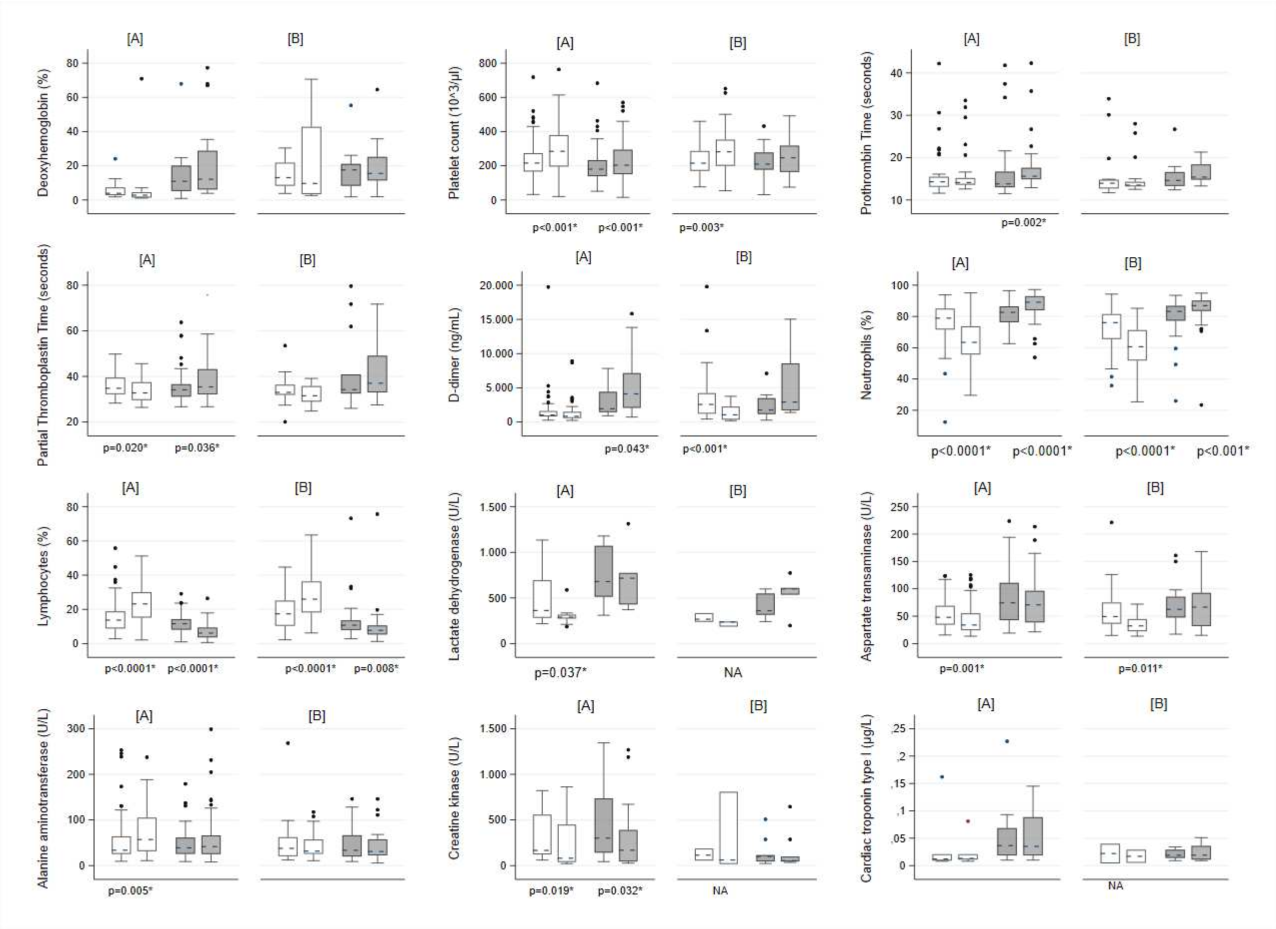

Figure S3. *Laboratory biomarkers' changes over time. In these boxplots are illustrated only patients whom data are available both at hospital admission and at discharge. Single graph legend: male patients on the left (the first four bars [A]), female on the right (second group of four bars, [B]). Within four-bars group: discharge live patients are white color couple of bars, grey color couple of bars is for died patients. Within couple of bars: first bar represents biomarker's values at hospital admission, second bar represents biomarker's values at hospital discharge. Example: First graph: first four-bars group, panel [A]: 1st bar: deoxyhemoglobin value at admission for discharged alive male patients, 2nd bar: deoxyhemoglobin value at discharge for discharged alive male patients, 3rd bar: deoxyhemoglobin value at admission for died male patients, 4th bar: deoxyhemoglobin value at discharge for died male patients. Second four-bars group, panel [B]: same as male is for female. Statistically significance is only reported if p<0.05, NA is for not applicable test because the low number of available data.*

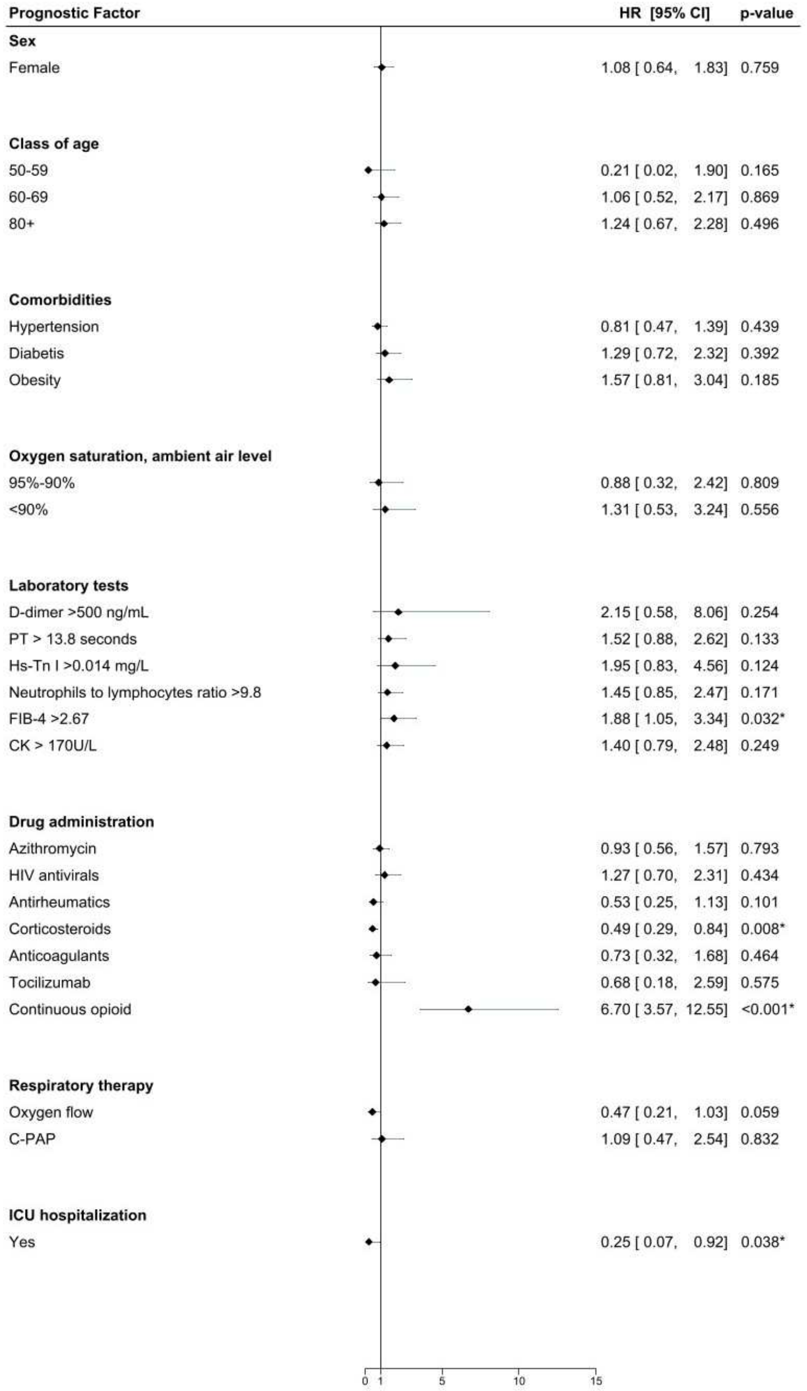

Figure S4. *Multivariable Cox PH regression model illustrated by a Forest Plot. Male sex, 70-79 class of age and >95% oxygen saturation level are the reference covariates. Legend: HR is for Hazard Ratio [95% confidence interval]; star symbol is for statistically significant at 0.05 level.*
